# Methyl 2-amino-4,5-di­meth­oxy­benzoate

**DOI:** 10.1107/S1600536813030894

**Published:** 2013-11-16

**Authors:** Tania N. Hill, Naadiya Patel

**Affiliations:** a Molecular Sciences Institute, School of Chemistry, University of the Witwatersrand, PO WITS 2050, Johannesburg, South Africa

## Abstract

The title compound, C_10_H_13_NO_4_, is essentially planar, with an r.m.s. deviation of 0.049 Å. An intra­molecular C—H⋯O hydrogen bond occurs and the amino group forms an intra­molecular N—H⋯O_ester_ hydrogen bond; the other H atom forms an inter­molecular N—H⋯O_carbon­yl_ hydrogen bond, leading to the formation of a helical chain that runs along the *b-*axis direction.

## Related literature

For similar crystal structures, see: Zhang *et al.* (2009 ▶); Smith & Elsegood (2002 ▶).
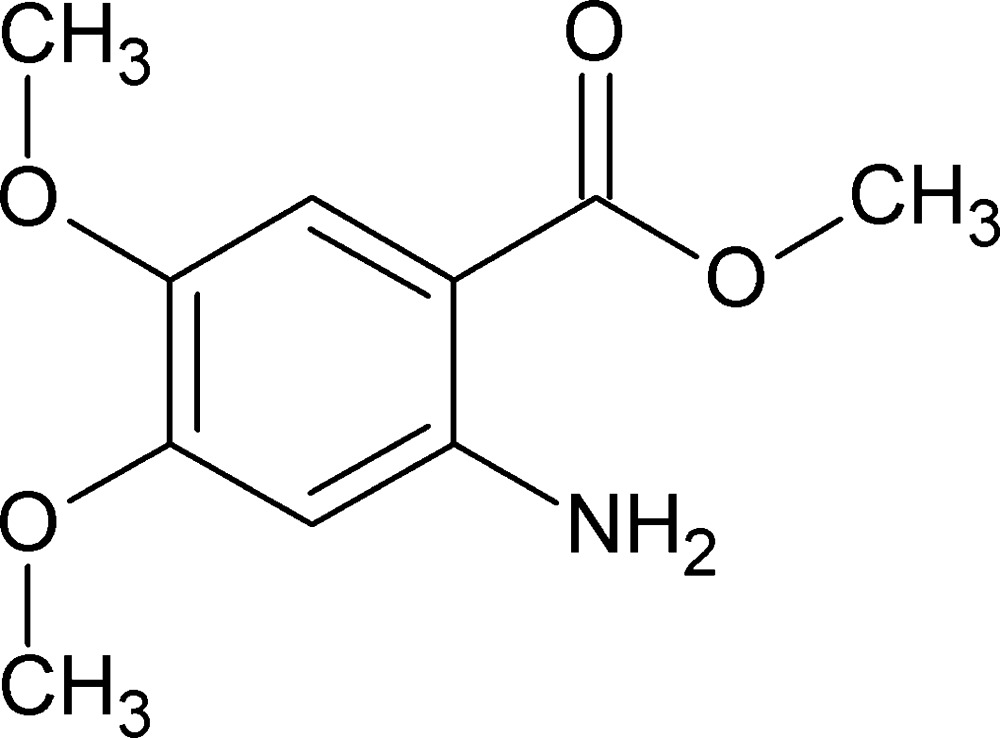


## Experimental

### 

#### Crystal data

C_10_H_13_NO_4_
*M* *_r_* = 211.21
Monoclinic, 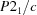

*a* = 11.1933 (4) Å
*b* = 7.7564 (3) Å
*c* = 13.7728 (5) Å
β = 121.741 (2)°
*V* = 1016.91 (7) Å^3^
*Z* = 4
Mo *K*α radiation
μ = 0.11 mm^−1^
*T* = 173 K
0.5 × 0.29 × 0.25 mm

#### Data collection

Bruker APEXII CCD area-detector diffractometer
Absorption correction: multi-scan (*SADABS*; Bruker, 2004 ▶) *T*_min_ = 0.948, *T*_max_ = 0.974
9994 measured reflections
2543 independent reflections
2080 reflections with *I* > 2σ(*I*)
*R*_int_ = 0.035

#### Refinement

*R*[*F* ^2^ > 2σ(*F* ^2^)] = 0.038
*wR*(*F* ^2^) = 0.109
*S* = 1.05
2543 reflections
147 parameters
H atoms treated by a mixture of independent and constrained refinement
Δρ_max_ = 0.27 e Å^−3^
Δρ_min_ = −0.25 e Å^−3^

### 

Data collection: *APEX2* (Bruker, 2005 ▶); cell refinement: *SAINT-Plus* (Bruker, 2004 ▶); data reduction: *SAINT-Plus* and *XPREP* (Bruker, 2004 ▶); program(s) used to solve structure: *SHELXS97* (Sheldrick, 2008 ▶); program(s) used to refine structure: *SHELXL97* (Sheldrick, 2008 ▶); molecular graphics: *DIAMOND* (Brandenburg & Putz, 2005 ▶); software used to prepare material for publication: *WinGX* (Farrugia, 2012) ▶.

## Supplementary Material

Crystal structure: contains datablock(s) global, I. DOI: 10.1107/S1600536813030894/ng5347sup1.cif

Structure factors: contains datablock(s) I. DOI: 10.1107/S1600536813030894/ng5347Isup2.hkl

Supplementary material file. DOI: 10.1107/S1600536813030894/ng5347Isup3.cml

Additional supplementary materials:  crystallographic information; 3D view; checkCIF report

## Figures and Tables

**Table 1:** Hydrogen-bond geometry (Å, °)

*D*—H⋯*A*	*D*—H	H⋯*A*	*D*⋯*A*	*D*—H⋯*A*
C6—H6⋯O2	0.95	2.37	2.7131 (14)	101
N1—H1*A*⋯O1	0.90 (2)	2.03 (2)	2.702 (2)	131 (2)
N1—H1*B*⋯O1^i^	0.88 (2)	2.10 (2)	2.947 (1)	162 (1)

Symmetry code: (i) 
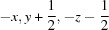
.

**Fig. 1.**
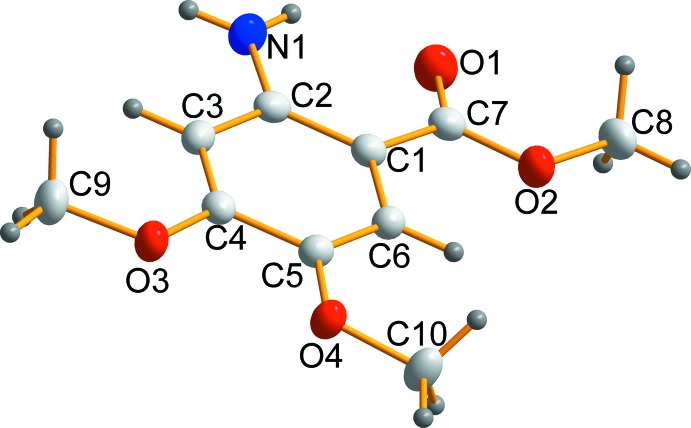
View of (I) (50% probability displacement ellipsoids)

**Fig. 2.**
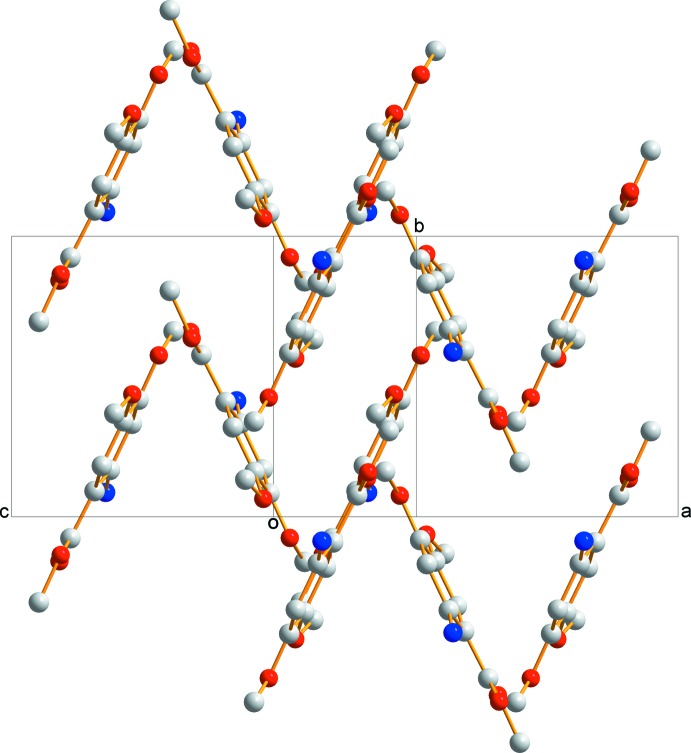
Packing of (I) viewed along [0 1 0]. H atoms omitted.

## supplementary crystallographic information

### 1. Comment

The molecular structure of (I) is presented in Figure 1, and was obtained by recrystallization of the commercially available compound. The title compound consists of an amino (C2) and methoxy (C4 and C5) substituted benzoate which was found to be essentially planar with an *r.m.s.* deviation of 0.049 Å; the dihedral angles shown in Table 1 further reinforce the planarity of (I). The maximum deviation observed below the calculated mean plane was found for the carbonyl oxygen (O1) at -0.136 (1) Å. Two intramolecular hydrogen bonds found between the phenyl carbon (C6), the amino (N1) and the ester O atoms (O1 and O2) with distances of 2.713 (1) Å and 2.702 (2) Å, effectively lock the ester into the molecular plane. The last hydrogen bond interaction observed between the amino (N1) and an adjacent carbonyl oxygen (O1) (symmetry operator [-*x*, *y* + 1/2, -*z* - 1/2]) with a distance of 2.947 (1) Å results in a helical chain along [0 1 0] (Figure 2).

### 2. Experimental

Methyl 2-amino-4,5-dimethoxybenzoate was obtained commercially. (I) was redissolved in warm MeOH and allowed to cool to room terperature. Yellow crystals suitable for single-crystal diffraction were obtained by slow evaporation over a few days.

### 3. Refinement

All hydrogen atoms were positioned geometrically and refined using a riding model, with C—H = 0.95 Å *U*_iso_(H)= 1.2 *U*_eq_(C) for the aromatic H and with C—H = 0.98 Å *U*_iso_(H)= 1.5 *U*_eq_(C)for methyl H atoms. The methyl groups were allowed to rotate with a fixed angle arround the C—C bond to best fit the experimental electron density. The amino hydrogens were freely refined.

### Crystal data

**Table:**

C_10_H_13_NO_4_	*F*(000) = 448
*M**_r_* = 211.21	*D*_x_ = 1.38 Mg m^−^^3^
Monoclinic, *P*2_1_/*c*	Mo *K*α radiation, λ = 0.71073 Å
Hall symbol: -P 2ybc	Cell parameters from 3834 reflections
*a* = 11.1933 (4) Å	θ = 3.0–28.3°
*b* = 7.7564 (3) Å	µ = 0.11 mm^−^^1^
*c* = 13.7728 (5) Å	*T* = 173 K
β = 121.741 (2)°	Cuboid, yellow
*V* = 1016.91 (7) Å^3^	0.5 × 0.29 × 0.25 mm
*Z* = 4	

### Data collection

**Table:**

Bruker APEXII CCD area-detector diffractometer	2543 independent reflections
Radiation source: fine-focus sealed tube	2080 reflections with *I* > 2σ(*I*)
Graphite monochromator	*R*_int_ = 0.035
Detector resolution: 512 pixels mm^-1^	θ_max_ = 28.3°, θ_min_ = 2.1°
ω scans	*h* = −14→14
Absorption correction: multi-scan (*SADABS*; Bruker, 2004)	*k* = −10→10
*T*_min_ = 0.948, *T*_max_ = 0.974	*l* = −18→18
9994 measured reflections	

### Refinement

**Table:**

Refinement on *F*^2^	0 restraints
Least-squares matrix: full	H atoms treated by a mixture of independent and constrained refinement
*R*[*F*^2^ > 2σ(*F*^2^)] = 0.038	*w* = 1/[σ^2^(*F*_o_^2^) + (0.0501*P*)^2^ + 0.2646*P*] where *P* = (*F*_o_^2^ + 2*F*_c_^2^)/3
*wR*(*F*^2^) = 0.109	(Δ/σ)_max_ < 0.001
*S* = 1.05	Δρ_max_ = 0.27 e Å^−^^3^
2543 reflections	Δρ_min_ = −0.25 e Å^−^^3^
147 parameters	

### Special details

**Table:**

Geometry. All e.s.d.'s (except the e.s.d. in the dihedral angle between two l.s. planes) are estimated using the full covariance matrix. The cell e.s.d.'s are taken into account individually in the estimation of e.s.d.'s in distances, angles and torsion angles; correlations between e.s.d.'s in cell parameters are only used when they are defined by crystal symmetry. An approximate (isotropic) treatment of cell e.s.d.'s is used for estimating e.s.d.'s involving l.s. planes.

### Fractional atomic coordinates and isotropic or equivalent isotropic displacement parameters (Å^2^)

**Table:**

	*x*	*y*	*z*	*U*_iso_*/*U*_eq_	
C1	0.19769 (12)	0.09141 (14)	−0.01177 (10)	0.0237 (2)	
C2	0.16865 (12)	0.16680 (14)	−0.11496 (10)	0.0243 (2)	
C3	0.22538 (12)	0.33134 (15)	−0.11104 (10)	0.0250 (2)	
H3	0.2062	0.3839	−0.1801	0.03*	
C4	0.30761 (12)	0.41704 (14)	−0.00949 (10)	0.0239 (2)	
C5	0.33814 (12)	0.34050 (14)	0.09484 (9)	0.0234 (2)	
C6	0.28295 (12)	0.18137 (14)	0.09202 (9)	0.0235 (2)	
H6	0.3024	0.1301	0.1615	0.028*	
C7	0.13820 (12)	−0.07648 (14)	−0.01151 (10)	0.0256 (2)	
C8	0.13103 (15)	−0.30520 (15)	0.09811 (12)	0.0340 (3)	
H8A	0.0291	−0.3006	0.064	0.051*	
H8B	0.1763	−0.3421	0.1779	0.051*	
H8C	0.1531	−0.3875	0.0558	0.051*	
C9	0.33489 (15)	0.66056 (17)	−0.10146 (11)	0.0345 (3)	
H9A	0.3706	0.591	−0.1402	0.052*	
H9B	0.3805	0.7738	−0.0826	0.052*	
H9C	0.2331	0.6751	−0.1518	0.052*	
C10	0.44564 (14)	0.36738 (17)	0.29532 (10)	0.0328 (3)	
H10A	0.3554	0.3526	0.29	0.049*	
H10B	0.5049	0.4467	0.3579	0.049*	
H10C	0.4927	0.2554	0.31	0.049*	
N1	0.09141 (12)	0.08643 (15)	−0.21889 (9)	0.0326 (3)	
O1	0.05611 (10)	−0.15847 (12)	−0.09659 (8)	0.0376 (2)	
O2	0.18202 (10)	−0.13617 (11)	0.09315 (7)	0.0324 (2)	
O3	0.36486 (9)	0.57535 (10)	0.00121 (7)	0.0293 (2)	
O4	0.42222 (9)	0.43624 (10)	0.19078 (7)	0.0279 (2)	
H1B	0.0560 (16)	0.1496 (19)	−0.2812 (14)	0.037 (4)*	
H1A	0.0410 (18)	−0.007 (3)	−0.2225 (15)	0.056 (5)*	

### Atomic displacement parameters (Å^2^)

**Table:**

	*U*^11^	*U*^22^	*U*^33^	*U*^12^	*U*^13^	*U*^23^
C1	0.0235 (5)	0.0231 (5)	0.0239 (5)	0.0032 (4)	0.0121 (4)	−0.0001 (4)
C2	0.0221 (5)	0.0261 (5)	0.0232 (5)	0.0050 (4)	0.0110 (4)	−0.0010 (4)
C3	0.0261 (6)	0.0278 (6)	0.0223 (5)	0.0056 (4)	0.0137 (5)	0.0038 (4)
C4	0.0248 (6)	0.0229 (5)	0.0268 (5)	0.0033 (4)	0.0155 (5)	0.0024 (4)
C5	0.0236 (5)	0.0249 (5)	0.0219 (5)	0.0017 (4)	0.0121 (4)	−0.0004 (4)
C6	0.0255 (6)	0.0237 (5)	0.0216 (5)	0.0021 (4)	0.0127 (4)	0.0011 (4)
C7	0.0250 (6)	0.0246 (5)	0.0272 (6)	0.0032 (4)	0.0137 (5)	−0.0011 (4)
C8	0.0438 (8)	0.0221 (5)	0.0417 (7)	−0.0015 (5)	0.0263 (6)	0.0015 (5)
C9	0.0402 (7)	0.0328 (6)	0.0336 (6)	0.0003 (5)	0.0214 (6)	0.0092 (5)
C10	0.0398 (7)	0.0348 (6)	0.0218 (6)	−0.0095 (5)	0.0148 (5)	−0.0019 (5)
N1	0.0371 (6)	0.0324 (6)	0.0211 (5)	−0.0008 (5)	0.0102 (5)	−0.0008 (4)
O1	0.0411 (5)	0.0323 (5)	0.0305 (5)	−0.0088 (4)	0.0127 (4)	−0.0055 (4)
O2	0.0431 (5)	0.0241 (4)	0.0295 (4)	−0.0050 (4)	0.0188 (4)	−0.0004 (3)
O3	0.0363 (5)	0.0258 (4)	0.0271 (4)	−0.0031 (3)	0.0176 (4)	0.0029 (3)
O4	0.0341 (5)	0.0271 (4)	0.0215 (4)	−0.0063 (3)	0.0140 (4)	−0.0018 (3)

### Geometric parameters (Å, º)

**Table:**

C1—C2	1.4077 (15)	C8—O2	1.4458 (14)
C1—C6	1.4162 (15)	C8—H8A	0.98
C1—C7	1.4634 (16)	C8—H8B	0.98
C2—N1	1.3722 (15)	C8—H8C	0.98
C2—C3	1.4138 (16)	C9—O3	1.4317 (14)
C3—C4	1.3751 (16)	C9—H9A	0.98
C3—H3	0.95	C9—H9B	0.98
C4—O3	1.3568 (13)	C9—H9C	0.98
C4—C5	1.4203 (15)	C10—O4	1.4247 (14)
C5—O4	1.3692 (13)	C10—H10A	0.98
C5—C6	1.3716 (15)	C10—H10B	0.98
C6—H6	0.95	C10—H10C	0.98
C7—O1	1.2202 (14)	N1—H1B	0.881 (16)
C7—O2	1.3374 (14)	N1—H1A	0.90 (2)
			
C2—C1—C6	119.31 (10)	H8A—C8—H8B	109.5
C2—C1—C7	120.56 (10)	O2—C8—H8C	109.5
C6—C1—C7	120.12 (10)	H8A—C8—H8C	109.5
N1—C2—C1	123.27 (11)	H8B—C8—H8C	109.5
N1—C2—C3	118.28 (10)	O3—C9—H9A	109.5
C1—C2—C3	118.42 (10)	O3—C9—H9B	109.5
C4—C3—C2	121.47 (10)	H9A—C9—H9B	109.5
C4—C3—H3	119.3	O3—C9—H9C	109.5
C2—C3—H3	119.3	H9A—C9—H9C	109.5
O3—C4—C3	124.97 (10)	H9B—C9—H9C	109.5
O3—C4—C5	114.83 (10)	O4—C10—H10A	109.5
C3—C4—C5	120.20 (10)	O4—C10—H10B	109.5
O4—C5—C6	125.87 (10)	H10A—C10—H10B	109.5
O4—C5—C4	115.28 (10)	O4—C10—H10C	109.5
C6—C5—C4	118.85 (10)	H10A—C10—H10C	109.5
C5—C6—C1	121.75 (10)	H10B—C10—H10C	109.5
C5—C6—H6	119.1	C2—N1—H1B	118.5 (10)
C1—C6—H6	119.1	C2—N1—H1A	117.0 (12)
O1—C7—O2	121.23 (11)	H1B—N1—H1A	116.4 (15)
O1—C7—C1	125.11 (11)	C7—O2—C8	115.77 (9)
O2—C7—C1	113.66 (10)	C4—O3—C9	117.30 (9)
O2—C8—H8A	109.5	C5—O4—C10	116.08 (9)
O2—C8—H8B	109.5		
			
C6—C1—C2—N1	177.57 (11)	C4—C5—C6—C1	0.54 (17)
C7—C1—C2—N1	−3.72 (17)	C2—C1—C6—C5	0.06 (17)
C6—C1—C2—C3	−0.46 (16)	C7—C1—C6—C5	−178.65 (10)
C7—C1—C2—C3	178.25 (10)	C2—C1—C7—O1	−3.96 (18)
N1—C2—C3—C4	−177.87 (11)	C6—C1—C7—O1	174.73 (11)
C1—C2—C3—C4	0.26 (16)	C2—C1—C7—O2	176.09 (10)
C2—C3—C4—O3	−179.49 (10)	C6—C1—C7—O2	−5.22 (15)
C2—C3—C4—C5	0.33 (17)	O1—C7—O2—C8	2.67 (16)
O3—C4—C5—O4	−0.88 (14)	C1—C7—O2—C8	−177.38 (10)
C3—C4—C5—O4	179.28 (10)	C3—C4—O3—C9	1.26 (16)
O3—C4—C5—C6	179.10 (10)	C5—C4—O3—C9	−178.57 (10)
C3—C4—C5—C6	−0.73 (17)	C6—C5—O4—C10	−4.40 (16)
O4—C5—C6—C1	−179.48 (10)	C4—C5—O4—C10	175.59 (10)

### Hydrogen-bond geometry (Å, º)

**Table:**

*D*—H···*A*	*D*—H	H···*A*	*D*···*A*	*D*—H···*A*
C6—H6···O2	0.95	2.37	2.7131 (14)	101
N1—H1*A*···O1	0.90 (2)	2.03 (2)	2.702 (2)	131 (2)
N1—H1*B*···O1^i^	0.88 (2)	2.10 (2)	2.947 (1)	162 (1)

Symmetry code: (i) −*x*, *y*+1/2, −*z*−1/2.
